# Abdominal Obesity May Play a Significant Role in inflammation Exacerbation in Polycystic Ovary Syndrome Patients

**DOI:** 10.5935/1518-0557.20230065

**Published:** 2023

**Authors:** Fatemeh Shirvanizadeh, Akram Eidi, Maryam Hafezi, Poopak Eftekhari-Yazdi

**Affiliations:** 1 Department of Biology, Science and Research Branch, Islamic Azad University, Tehran, Iran; 2 Department of Endocrinology and Female Infertility, Reproductive Biomedicine Research Center, Royan Institute for Reproductive Biomedicine, ACECR, Tehran, Iran; 3 Department of Embryology, Reproductive Biomedicine Research Center, Royan Institute for Reproductive Biomedicine, ACECR, Tehran, Iran

**Keywords:** polycystic ovary syndrome, abdominal obesity, inflammation, tumor necrosis factor-α

## Abstract

**Objective:**

Polycystic ovary syndrome (PCOS) is an endocrine disorder that seems to be pro-inflammatory at many levels, abdominal obesity (AO) is a prevalent pro-inflammatory phenotype in PCOS patients, and it seems to contribute to the initiation or worsening of inflammation in PCOS patients. In this study, we investigated the role of the AO phenotype in the occurrence of other obesity indicators (neck and arm) and augmentation of inflammation in the follicular fluid (FF) of PCOS patients.

**Methods:**

40 patients under the age of 35 were divided into four groups: PCOS with AO, PCOS without AO, non-PCOS with AO, and non-PCOS without AO. The FF samples were collected from each patient. Clinical and anthropometric characteristics of the participants, as well as tumor necrosis factor-α (TNF-α) concentration in the FF samples, were quantitatively assessed using enzyme-linked immunosorbent assays. The number of retrieved cumulus-oocyte complexes (COC) and their quality were scored.

**Results:**

The PCOS^+^AO^+^ group had significantly increased neck circumference, compared to the other groups (*p*<0.001). The concentration of TNF-α was significantly higher in the PCOS+AO+ group than in the other groups (*p*<0.001). There were no significant differences in the number of retrieved COC per patient and the quality of oocytes between the groups (*p*>0.05).

**Conclusions:**

Given the significant role of inflammation in the development of PCOS, managing AO in PCOS patients may aid in reducing inflammation and could potentially help in the design of customized treatment approaches.

## INTRODUCTION

Polycystic ovary syndrome (PCOS) is one of the most common endocrine disorders and reproductive abnormalities among women of reproductive age, with a prevalence ranging from 5-18% (Costello *et al*., 2019; Özer *et al*., 2016; Zuo *et al*., 2016). The Rotterdam criteria were created to confirm diagnosis of PCOS in women who have at least two of the following symptoms; hyperandrogenism, polycystic ovaries and oligo- and/or anovulation (Broekmans *et al*., 2006). PCOS patients have metabolic disorders such as dyslipidemia, insulin resistance, pancreatic beta cell dysfunction, endothelial dysfunction, early onset of type 2 diabetes, lipid profile disorders, and visceral obesity, all of which affect health and fertility (Rocha *et al*., 2019). Other factors such as obesity, lifestyle, and genetic and environmental factors can contribute to the development of PCOS (Xie *et al*., 2019), associated with anovulation, which stimulates excess androgen synthesis and chronic inflammation due to ovarian dysfunction (Özer *et al*., 2016). Pro-inflammatory mediators in PCOS patients can disrupt insulin release and stimulate the production of androgens through ovarian theca cells, playing an essential role in inducing hyperandrogenism (HA) (Al-Jefout *et al*., 2017; Bongrani *et al*., 2022). Low-grade chronic inflammation has emerged as the main cause of long-term adverse outcomes in the pathogenesis of polycystic ovary syndrome (Rojas *et al*., 2014).

Recent reports have it that obesity plays a functional role in the pathogenesis of PCOS and has an increasing effect on developing metabolic disorders in PCOS patients. Approximately 60-70% of PCOS patients have abdominal obesity (AO), even normal-weight PCOS patients have excess visceral obesity and this adipose tissue may contribute to inflammation in these patients (Al-Jefout *et al*., 2017; Nasiri *et al*., 2015). Of course, this information is about abdominal obesity, and information about other indicators of obesity (neck and arm) with increased inflammation in PCOS patients is not available. AO induces an inflammatory response and reproductive disorders in PCOS patients via the secretion of inflammatory cytokines and the activation of nuclear factors (NF- kB) (Kałużna *et al*., 2020). This activation is related to the increase in the expression of pro-inflammatory mediators such as tumor necrosis factor-α (TNF-α) or interleukin-6 (IL-6) (Nehir Aytan *et al*., 2016). Physiologically, pro-inflammatory cytokines are produced during follicular development and are involved in ovulation induction, but long-term chronic inflammation can impair follicle development and ultimately cause adverse reproductive outcomes (Boots & Jungheim, 2015; Liu *et al*., 2021). It appears that AO as a potential trigger of PCOS greatly aggravates the pathogenic symptoms (Möhlig *et al*., 2004).

The follicular fluid (FF) and serum of PCOS patients have a high level of inflammatory markers such as interleukin-1 beta (IL-1β), tumor necrosis factor- α (TNF-α), and interleukin-6 (IL-6) (Szczuko *et al*., 2016). Serum and FF concentrations of TNF-α are also elevated in PCOS patients (Gaafar *et al*., 2014). TNF-α is a multifaceted cytokine secreted by macrophages and causes the proliferation of granulosa cells and changes in ovarian function (Gaafar *et al*., 2014; Prins *et al*., 2020). This cytokine is overexpressed in adipose tissue and causes insulin sensitivity. The expression of this cytokine is increased in obesity and plays an essential role in causing low-grade chronic inflammation and metabolic syndrome disorders (Spritzer *et al*., 2015).

Considering that AO is a pro-inflammatory phenotype, it seems that it can play a significant role in exacerbating inflammation in polycystic ovary syndrome (PCOS), which is a low-grade inflammatory disease, and can help to explain infertility in PCOS patients. In the present study, we investigated the specific role of the abdominal obesity (AO) phenotype in the occurrence of other obesity indicators (neck and arm) and the exacerbation of FF inflammation among PCOS patients. For this purpose, pro-inflammatory cytokine TNF-α concentrations in the FF samples of PCOS patients were evaluated by an enzyme-linked immunosorbent assay technique.

## MATERIALS AND METHODS

### Patients

The medical ethical committee of the Royan Institute (Tehran, Iran) approved the study (Ethical code: NO.IR. ACECR.ROYAN.REC.1400.091) and written informed consent was obtained from the patients. Forty patients (25-35 years old) with *in vitro* fertilization (IVF) or intracytoplasmic sperm injection (ICSI) with symptoms at least three months have passed since their last ovulation stimulation, body mass index (BMI) >30, without systemic inflammatory diseases, diabetes, hypertension (Solak *et al*., 2016) and thyroid disorders (Sniezek & Francis, 2003), without continuous use of anti-inflammatory drugs (at least 6-8 weeks before the start of the study) were included in our study. Patients with ovarian hyperstimulation syndrome (OHSS) and poor response to ovarian stimulation were considered as exclusion criteria.

### Study Design

In this observational cross-sectional study from October 2021 to June 2022, the forty patients were referred to the Royan Institute (Tehran, Iran), at the start of the IVF/ ICSI cycle and were divided into four groups based on the presence of PCOS (diagnosed by the Rotterdam 2004 criteria) or absence of PCOS (normal oogenesis women with a history of male factor, tubal factor, or egg donation), and presence or absence of AO (waist/hip ratio ≥ 0.80):

Group 1: Women with PCOS; with abdominal obesity (PCOS^+^AO^+^)

Group 2: Women with PCOS; without abdominal obesity (PCOS^+^AO)

Group 3: Non-PCOS women; with abdominal obesity (PCOS^-^AO^+^)

Group 4: Non-PCOS women; without abdominal obesity (PCOS^-^AO).

### Ovarian Stimulation

In accordance with the following antagonist protocol, all the study patients were treated with standard controlled ovarian stimulation and oocyte retrieval.

Before each patient’s entered the cycle, basic information including antral follicle count (AFC), anti-Müllerian hormone (AMH), luteinizing hormone (LH), and follicle-stimulating hormone (FSH) was evaluated. Controlled ovarian stimulation (COS), was started on day 2 or 3 of the cycle to ovulation induction. During the first 6 days of the menstrual cycle, the patients received regular, daily subcutaneous (SC) injections of 150 IU of the recombinant human follitropin-b (rFSH) (Puregonw, MSD, Ballerup, Denmark). On day 6, the serial vaginal ultrasonography was performed and based on the patient’s ovarian response when two or three ovarian follicles reached a diameter of ≥13 mm, patients received daily SC injections containing 0.25 mg of a GnRH antagonist (Ganirelix-Orgalutranw; MSD, Ballerup, Denmark). Then ovarian stimulation continued with rFSH along with the antagonist until the patients had at least more than three follicles with an average diameter of 17-18 mm and E2 levels of 1000-3500 pg/mL and the number of follicles on both sides should be less than or equal to 15 follicles and not had a risk of OHSS, received an SC injection of 6500IU or 13000 IU dosage of recombinant human chorionic gonadotrophin (rhCG) (Ovitrellew; Merk Serono, Hellerup, Denmark) to induce final oocyte maturation. And patients whose estradiol level was more than 3500 IU or high-risk patients for OHSS with more than 16 follicles were excluded from the study and GnRH agonists were used for all of them and all were frozen. A standard ultrasonically guided follicular puncture was used to retrieve oocytes 36 to 38 hours after hCG injection. Subsequently, these patients underwent IVF / ICSI process.

### Collecting Follicular Fluid Sample

Follicular fluid was taken from the dominant follicles (18-24 mm) on the day of oocyte retrieval. The collected liquid was centrifuged for 15 minutes at a speed of 1200 rpm until the follicular cells settled and separated supernatants, and then were filtered (0.22 µm pore size). All the samples were heat-inactivated at 56°C for 30 minutes. Then the accumulated supernatants were kept at -80°C until biochemical analysis.

### Oocyte maturity

Cumulus-oocyte complexes (COC) were retrieved 36 to 38 hours after the injection of hCG. The evolution of oocyte quality was performed via morphological assessment. The oocytes were graded into two groups: Metaphase II (MII) oocytes with normal morphology (round with a smooth first polar body, dispersed cytoplasmic granules, normal perivitelline space, homogeneous fine granularity, and zona pellucida thickness (18 µm)) as good oocytes and those with intracytoplasmic (vacuolization, accumulating saccules of smooth endoplasmic reticulum, organelle clustering) and/ or extracytoplasmic anomalies (large perivitelline space, abnormal and dark zona pellucida, granule in perivitelline space) as fair oocytes.

Fertilization was assessed 17 hours after *in vitro* fertilization (IVF) or intracytoplasmic sperm injection (ICSI). Embryos were transferred to culture medium up to embryo transfer day.

### TNF-α Concentration in Follicular Fluid Samples

According to the manufacturer’s protocol (CN: KPG-HT-NF-α48; pg/ml; LOT: HTNF0422004 Iran); TNF-α concentration was quantitatively investigated as an inflammatory marker in FF samples in all groups by enzyme-linked immunosorbent assays (ELISA).

### Statistical Analysis

Data were reported as mean ± SD, and the graphs were plotted using the GraphPad Prism program (virgin 9). The data were statistically analyzed using analysis of the Kolmogorov–Smirnov test, and variances (Two-way ANOVA) followed by a post-Tukey test, and a *p-value* <0.05 was considered a significant difference.

## RESULTS

### Clinical and hormonal findings

A total of 40 follicular fluid samples from candidates with and without PCOS, who were undergoing oocyte retrieval for IVF/ICSI before ovarian stimulation and in the clinical laboratory of Royan Institute (Tehran, Iran) were evaluated. Table 1 shows the characteristics of the women in the four groups of the study. Clinical factors, including age, luteinizing hormone/follicle-stimulating hormone ratio, body mass index, thyroid stimulating hormone, and prolactin, did not significantly differ among the four groups (*p>*0.05), but free testosterone (as a biomarker of hyperandrogenism) and anti-müllerian hormone in PCOS^+^AO^+^ group were significantly higher compared with others (*p*<0.001).

**Table 1:** Clinical characteristics in studied groups classified according to PCOS and AO.

Patients (n)	PCOS^+^AO^+^ (n=10)	PCOS^+^AO^-^(n=10)	PCOS^-^AO^+^ (n=10)	PCOS^-^AO^-^(n=10)	*p* value
Age (Years)	28.8±3.32	28.25±2.25	29.7±3.65	29.91±4.71	0.742
BMI (Kg/M^2^)	25.99±3.65	20.74±7.81	26.65±3.31	23.22±7.93	0.167
LH/FSH (mIU/ml)	1 .65±0.49	1.42±0.78	0.766±0.33	0.87±0.54	0.078
AMH (ng/ml)	5.83±3.33*, ^†^	5.96±2.07 ^‡,^^§^	3.25±1.13*, ‡	2.83±0.93 ^†,^^§^	0.001
TSH (mIU/ml)	3.08±1.45	2.63±1.66	1.91±1.28	1.96±0.93	0.445
PRL (IU/L)	187.5±33.68	182.6±68.75	166.2±46.66	152.8±50.44	0.535
Free testosterone (ng/ml)	1.77±0.43*, ^†^	1.29±0.37	0.99±0.47*	0.81±0.36 ^†^	<0.001

Data are mean ± SD; PCOS: Polycystic ovary syndrome; AO: abdominal obesity; BMI: body mass index; Similar superscripts indicate a statistically significant difference (*p*<0.001).

### Anthropometric findings

Table 2 shows the arm and neck circumference as upper body obesity index. Neck circumference was significantly increased in the PCOS^+^AO^+^ group compared with others (*p*<0.001).

**Table 2:** Anthropometric characteristics in studied groups classified according to PCOS and AO.

	PCOS^+^AO^+^ (n=10)	PCOS^+^AO^-^(n=10)	PCOS^-^AO^+^ (n=10)	PCOS^-^AO^-^(n=10)	*p* value
WHR (cm/cm)	0.87±0.51*,^†,^^¶^	0.75±0.25*^,‡,¶^	0.85±0.41^‡,§^	0.76±0.25^†,^^§^	<0.001
Neck circumference (cm)	35.3±1.63*^,†^	32.25±1.66*, ‡	35.00±2.00^‡,§^	32.25±2.41^†,§^	0.001
Arm circumference (cm)	33.1±3.41	29.37±3.11	31.20±2.09	31.16±4.66	0.141

Data are mean ± SD; PCOS: Polycystic ovary syndrome; AO: abdominal obesity; WHR: waist/hip ratio; Similar superscripts indicate a statistically significant difference (*p*<0.001).

### TNF α levels

**Figure 1:**
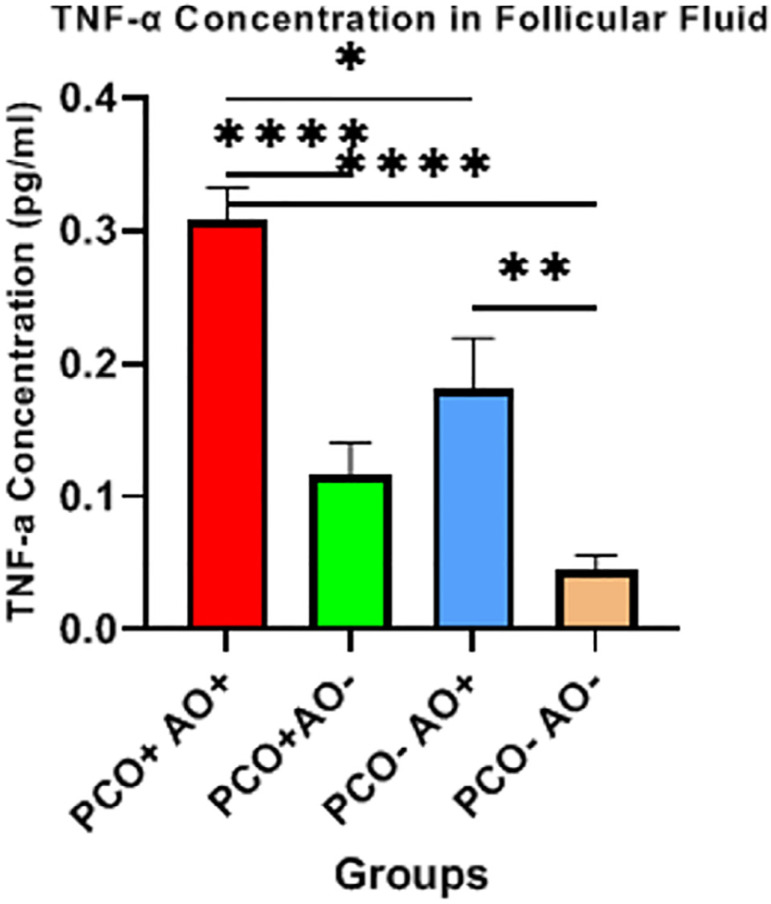
The concentration of TNF-α in follicular fluid in four groups was measured by ELISA Data are mean ± SEM; n=10 per group; *p<0.05, **p<0.01, ****p<0.0001 were considered statistically significant.

The concentration of TNF-α as pro-inflammatory cytokine was considerably higher in the PCOS^+^AO^+^ group compared to the others (*p*<0.001) (Figure 1).

### Quality of oocytes

The number of retrieved COC per patient and quality of oocytes did not significantly differ between groups (*p>*0.05) (Table 3).

**Table 3:** Anthropometric characteristics in studied groups classified according to PCOS and AO.

	PCOS^+^AO^+^ (n=10)	PCOS^+^AO^-^(n=10)	PCOS^-^AO^+^ (n=10)	PCOS^-^AO^-^(n=10)	*p* value
Number of retrieved COC	**10.1±4.66**	11±3.81	10±3.94	12.16±4.50	0.608
Oocytes quality (%)	Good: 10% Fair: 90%	Good: 25% Fair: 75%	Good: 20% Fair: 80%	Good: 41.7% Fair: 58.3%	0.683

Data are mean ± SD; PCOS: Polycystic ovary syndrome; AO: abdominal obesity; COC: Cumulus Oocyte Complex; Good oocytes: MII Oocytes with normal morphology; Fair oocytes: MII Oocytes with intra and/or extra cytoplasmic abnormalities.

## DISCUSSION

Metabolic abnormalities such as hyperinsulinemia, insulin resistance, dyslipidemia, and obesity are often present in PCOS women (Liu *et al.,* 2022). Recent studies have used the measurement of the waist-to-hip ratio of PCOS patients as a simple, cheap, non-invasive, available method to establish the amount of visceral obesity. Measuring the neck and arm circumference in PCOS patients as an anthropometric parameter representing the upper body’s subcutaneous adipose tissue can also be an innovative tool for screening abdominal obesity distribution, which reflects abdominal obesity and metabolic disorders (Liu *et al.,* 2022; Yang *et al.,* 2021). The results of the present study also show that patients with excessive adiposity (AO) have more upper-body obesity (around the neck and arms), which worsens hyperandrogenism. Of course, arm obesity was not significant, probably due to the small sample size in our study, but studies with larger sample sizes need to verify this.

The results of the study show that the TNF-α in the FF of PCOS patients with AO is significantly higher than in the other groups. This result is consistent with several other studies that reported high levels of pro-inflammatory cytokines in PCOS patients (Adams *et al.,* 2016; Amato *et al.,* 2003; Liu *et al.,* 2021; Zhang *et al.,* 2017). Most researchers consider inflammation as a key characteristic in PCOS patients and PCOS as a chronic inflammatory disease (Ghowsi *et al*., 2018). Although the exact mechanism is not fully understood yet, studies have shown that inflammation in these patients is caused by an increase in androgen synthesis by the ovary, which by stimulating androgen secretion, causes ovarian and adrenal hyperandrogenism (Repaci *et al*., 2011). The increase in androgens hinders the synthesis of sex hormone-binding globulin (SHBG), raises blood glucose levels, and leads to the accumulation of fat in the abdominal area. Consequently, it disrupts the normal menstrual cycle, interferes with follicular maturation, and contributes to the development of complications associated with PCOS (Nehir Aytan *et al*., 2016; Rudnicka *et al*., 2021).

Inflammation resulting from reduced expression of the glucose transporter gene type 4 (GLUT4) and excessive production of TNF-α in adipose tissue leads to insulin resistance (Samy *et al*., 2009). The phosphorylation of insulin receptor substrate- 1 (IRS-1) by intracellular serine kinases leads to the disruption of signaling events and decreased insulin sensitivity (Ghowsi *et al*., 2018). In PCOS patients, the balance of pro-inflammatory cytokines such as TNF-α, IL-6, and IL-18 and anti-inflammatory cytokines such as IL-27, IL-35, and IL-37 is disturbed, and the levels of pro-inflammatory cytokines increases dramatically. As a consequence, the ovulation process is disrupted due to the increase in unregulated inflammation, leading to infertility (Ghowsi *et al*., 2018).

The buildup of adipose tissue exacerbates the inflammation seen in these patients (Boots & Jungheim, 2015). Adipose tissue plays a crucial role in regulating glucose and lipid metabolism, which can affect energy consumption, inflammation, and cardiovascular and reproductive functions. Adipose tissue releases various cytokines, acute phase proteins, and other inflammatory mediators, which can have autocrine, paracrine, or systemic effects that impact glucose metabolism, energy balance, and proin-flammatory or anti-inflammatory activities (Coelho *et al*., 2013). Abdominal obesity (AO) is prevalent in 38-88% of PCOS patients, leading to insulin resistance by inhibiting insulin receptor tyrosine kinase in fat muscles (Oróstica *et al*., 2016). Obese women with PCOS experience high levels of free testosterone, androgens, insulin resistance, and a relative increase in blood sugar compared to women with normal weight, which leads to infertility, frequent miscarriages, menstrual and ovulation abnormalities, type 2 diabetes, high blood pressure, and implantation problems include decreasing the implantation rate (Velez *et al*., 2021).

Hyperandrogenism as an inflammation trigger can be independent of obesity or associated with excessive AO (Nehir Aytan *et al*., 2016). Studies have shown that hyper-androgenism exists in both obese and lean PCOS patients; but obesity, especially AO can increase hyperandrogenism (Mohammadi *et al*., 2017; Velez *et al*., 2021). Excessive androgens can cause hypertrophy of fat cells, leading to hypoxia, production of reactive oxygen species, and fat cell necrosis. In addition, fat tissue can activate the nuclear factor NF-κB (an inflammatory factor) by inducing oxidative stress and reducing antioxidant capacity, resulting in an increase in inflammatory cytokines such as TNF-α (Velez *et al*., 2021). There is thus a close correlation between inflammation, obesity, hyperinsulinemia, hyperandrogenism, and PCOS; they are interconnected and reinforce each other through several signaling pathways (Thathapudi *et al*., 2014).

In conclusion, the buildup of visceral adipose tissue is a crucial factor that contributes to the metabolic syndrome features associated with PCOS and chronic low-grade inflammation (Mohammadi *et al*., 2017). inflammatory cytokines play an important role in the proliferation of follicular theca cells, in the development of chronic low-grade inflammation, cancer, and the regulation of ovarian activity during the menstrual cycle. These cytokines are considered to be the primary candidates in molecular events, activation, and regulation of pro-inflammatory cascade in PCOS patients (Mohammadi *et al*., 2017). Furthermore, excessive production of TNF-α by fatty tissue can disrupt insulin function in various cells, including endothelial, epithelial, fibroblast, and endometrial tissue, especially in PCOS patients, and impairs their reproductive function (Rostamtabar *et al*., 2021).

In addition to the mild pro-inflammatory environment generated in the endometrium of these women, obesity also creates higher levels of inflammation in the endometrium of obese patients with PCOS. The current study showed that the level of TNF-α expression was highest in PCOS patients with AO compared with other groups, consistent with the results of previous studies. Consequently, the expressions of inflammatory factors in the serum and FF of PCOS patients are greatly increased, disrupting the ovulatory process and successful fertility in these patients (Oróstica *et al*., 2016).

Inflammation plays a key physiological role in folliculogenesis and ovulation, and an unperturbed inflammatory response is essential for proper folliculogenesis. Mounting evidence suggests that abnormal inflammation can disrupt normal ovarian follicle dynamics and result in impaired oocyte quality. Failure to ovulate and decreased implantation can result in infertility (Boots & Jungheim, 2015).

**Figure 2:**
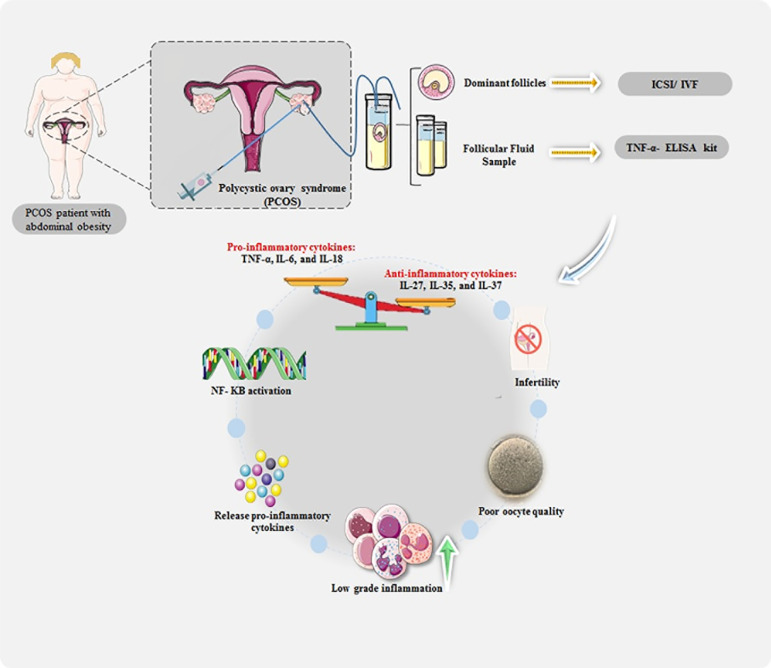
The schematic illustration of the association between AO and inflammation in PCOS patients in decreasing fertility potential.

The findings of the current study show that the pro-inflammatory state of PCOS plays an essential role in causing the complications of this disease. In addition, AO associated with PCOS significantly increases inflammatory status and hyperandrogenism (Liu *et al*., 2021). The results of our study were consistent with Niu’s finding that increased inflammation in the group of PCOS with AO was associated with a decrease in the number of good-quality oocytes (Niu *et al*., 2017) (Figure 2).

The combination of visceral obesity and chronic inflammation in PCOS patients likely leads to a disruption of oocyte quality and a significant reduction in fertility in obese PCOS women. inflammatory changes in women with PCOS may have a crucial role in drug approaches, treatment response, and metabolic and reproductive impairments in women with this syndrome.

## CONCLUSION

Considering the role of AO in aggravating hyperandrogenism and subsequently increasing pro-inflammatory cytokine levels and inducing inflammation, more patients should be included in the study, and if the same results are repeated, it is recommended that a new therapeutic protocol based on lifestyle improvement (including diet therapy, exercise, and weight loss) and pharmacotherapy should be selected for these patients before entering the treatment cycle.
